# HEARTSMAP-U: Adapting a Psychosocial Self-Screening and Resource Navigation Support Tool for Use by Post-secondary Students

**DOI:** 10.3389/fpsyt.2022.812965

**Published:** 2022-02-22

**Authors:** Punit Virk, Ravia Arora, Heather Burt, Anne Gadermann, Skye Barbic, Marna Nelson, Jana Davidson, Peter Cornish, Quynh Doan

**Affiliations:** ^1^School of Population and Public Health, Faculty of Medicine, University of British Columbia, Vancouver, BC, Canada; ^2^BC Children's Hospital Research Institute, Vancouver, BC, Canada; ^3^Centre for Health Evaluation and Outcome Sciences, Providence Health, Vancouver, BC, Canada; ^4^Department of Occupational Science and Occupational Therapy, Faculty of Medicine, University of British Columbia, Vancouver, BC, Canada; ^5^Student Health Service, University of British Columbia, Vancouver, BC, Canada; ^6^Division of Child and Adolescent Psychiatry, Department of Psychiatry, Faculty of Medicine, University of British Columbia, Vancouver, BC, Canada; ^7^Student Counselling Services, University of California, Berkeley, Berkeley, CA, United States; ^8^Division of Emergency Medicine, Department of Pediatrics, Faculty of Medicine, University of British Columbia, Vancouver, BC, Canada

**Keywords:** mental health, screening, validity, post-secondary students, focus groups

## Abstract

**Background:**

Mental health challenges are highly prevalent in the post-secondary educational setting. Screening instruments have been shown to improve early detection and intervention. However, these tools often focus on specific diagnosable conditions, are not always designed with students in mind, and lack resource navigational support.

**Objective:**

The aim of this study was to describe the adaptation of existing psychosocial assessment (HEARTSMAP) tools into a version that is fit-for-purpose for post-secondary students, called HEARTSMAP-U.

**Methods:**

We underwent a three-phase, multi-method tool adaptation process. First, a diverse study team proposed a preliminary version of HEARTSMAP-U and its conceptual framework. Second, we conducted a cross-sectional expert review study with Canadian mental health professionals (*N* = 28), to evaluate the clinical validity of tool content. Third, we conducted an iterative series of six focus groups with diverse post-secondary students (*N* = 54), to refine tool content and language, and ensure comprehensibility and relevance to end-users.

**Results:**

The adaptation process resulted in the HEARTSMAP-U self-assessment and resource navigational support tool, which evaluates psychosocial challenges across 10 sections. In Phase two, clinician experts expressed that HEARTSMAP-U's content aligned with their own professional experiences working with students. In Phase three, students identified multiple opportunities to improve the tool's end-user relevance by calling for more “common language,” such as including examples, definitions, and avoiding technical jargon.

**Conclusions:**

The HEARTSMAP-U tool is well-positioned for further studies of its quantitative psychometric properties and clinical utility in the post-secondary educational setting.

## Introduction

In recent years, post-secondary students have reported increasing levels of mental health challenges including psychological distress and diagnosed conditions (e.g., anxiety, depression) (1). While the post-secondary years are often a period of self-exploration and interpersonal growth (2), they have also been associated with high stress, peer pressure, and greater responsibilities with reduced social supports (3, 4). For young adults, this period coincides with significant physiological, psychological, and social development (5, 6). In 2019, Canadian data from the National College Health Assessment (*N* = 55,284) showed that, within the last 12-months, most post-secondary students reported experiencing overwhelming anxiety (68.9%) and at least half reported functionally impairing depression (51.6%) (7). Among the sample, 16.4% of students endorsed active suicidal ideation in the last 12-months, compared to 2.5% of the general Canadian adult population and 6% of young adults (ages 15–24 years) that same year (8, 9). During the COVID-19 pandemic, the rate of mental health concerns escalated in the student population, one study (*N* = 1,388) reported a 30-day anxiety and/or depressive symptom prevalence of 75% among Canadian students during the pandemic's first-wave (up till May 2020) (10). Similarly, the Healthy Minds survey (*N* = 18,764) saw increased prevalence of depression and lower levels of resiliency among American students compared to pre-pandemic estimates (11). The pandemic has compounded psychological and social challenges (psychosocial stressors) (12, 13), magnifying an already severe campus mental health crisis (14).

Students experience individual- and system-level barriers that may impede timely access to age-appropriate care. Low mental health literacy, poor system navigation support, and service saturation (e.g., wait-times) all impede help-seeking (15–18). National Canadian standards for student mental health and well-being call for institutions to have early identification and preventative infrastructures (19), which can improve long-term mental health outcomes and timely connectedness into services (20). Universal mental health screening and navigational support tools can address challenges institutions experience with identifying mental health concerns and supporting connectivity to care. Such measures have been successfully integrated within post-secondary health systems (21–23). Digital screening tools may alleviate the need for in-person intake assessment/triaging and more seamlessly bridge in-person and digital resources (3, 24, 25). Digital self-reporting of psychosocial challenges also shows higher disclosure rates and may be preferred over clinician-administered or paper-based assessment (26–28), offering users privacy, time, and space to articulate needs.

Notwithstanding the potential of screening, existing scales often focus exclusively on common psychological issues, such as the PHQ-9 (depression), GAD-7 (anxiety), AUDIT (substance use), and SBQ-R (suicidality) (29–32). These tools are diagnoses-specific, have not been developed with student engagement, and generally lack comprehensive validity evidence in student populations (33–36). However, several instruments have been developed or adapted with students' unique contextual (e.g., academic stress, social autonomy) and clinical needs (e.g., emerging adulthood) in mind. Downs et al. (37) previously developed the 34-item Symptoms and Assets Screening Scale specifically for college students to self-screen on common mental health challenges (e.g., eating disorder, substance abuse, anxiety, depressive symptoms) and generalized distress (37). Similarly, Alschuler et al. (38) developed the 11-item College Health Questionnaire, which facilitates behavioral screening of psychological (e.g., anxiety, depression) and social concerns (e.g., academic problems, relationships, finances) (38). Other post-secondary-specific screening and assessment measures include the Counseling Center Assessment of Psychological Symptoms and the Mental Health Continuum model. However, these assessment tools lack an actionable, resource navigational component, which may support students' help-seeking and contribute to the utility of screening (39–41).

Our team has previously developed, validated, and implemented psychosocial instruments for the pediatric population. The clinical HEARTSMAP assessment and management guiding tool supports pediatric acute care providers with psychosocial interviewing and disposition planning (42). MyHEARTSMAP is a self-administered version allowing self-/proxy-screening, to facilitate universal screening by youth and parents (43). Both instruments have demonstrated evidence for strong psychometric properties (42–45), high clinical utility (46, 47), and user acceptability (48). These instruments expand on the seminal HEADSS psychosocial interview and history-taking tool (49, 50) and assess ten broad psychosocial sections: Home, Education and activities, Alcohol and drugs, Relationships and bullying, Thoughts and anxiety, Safety, Sexual Health, Mood, Abuse, and Professional resources. These psychosocial issues are clinically significant and theoretically supported within human development and socio-ecological models. According to Maslow's Hierarchy of Needs, individuals work up from physiological (e.g., Home, Safety) and psychological needs (e.g., “Relationships”) toward self-fulfillment-oriented needs (e.g., “Education and activities) (51, 52). Within socio-ecological models, these psychosocial areas demonstrate how youths' mental well-being is shaped through the interplay of individual (e.g., Mood, Thoughts and anxiety, Safety risk), interpersonal (e.g., Relationships, Abuse, Sexual Health), institutional (e.g., Education and activities), and community factors (e.g., Professionals and resources) (19, 53, 54). We provide further details on the HEARTSMAP tools' measurement model, assessment structure, and resource recommendation decision-making algorithm in Web-Appendix A.

Adapted specifically for post-secondary students, HEARTSMAP-U is a brief, digital self-administered psychosocial screening tool. Similar to previous HEARTSMAP versions, HEARTSMAP-U assesses ten psychosocial areas ranging from Housing to Abuse. For each section, students first score their concerns on a 4-point Likert-type scale ranging from 0 (no concern) to 3 (severe concern), using anchor descriptions for each scoring option. Second, student's score whether they have previously accessed services pertaining to this section (yes/no). After students have answered these questions for all 10 sections, their responses feed into a built-in algorithm, triggering urgency-specific resource recommendations for identified mental health needs (13, 16, 19).

The current paper describes the three-phase process by which previously developed HEARTSMAP tools were adapted into HEARTSMAP-U, a version that is fit-for-purpose for the post-secondary student population, to help students self-identify psychosocial support needs. Our study will serve as a foundational paper on the HEARTSMAP-U tool and its preliminary adaptation. We will collect multi-faceted evidence of instrument validity and reliability in an ongoing manner and report it in later studies.

## Methods

**Figure 1:**
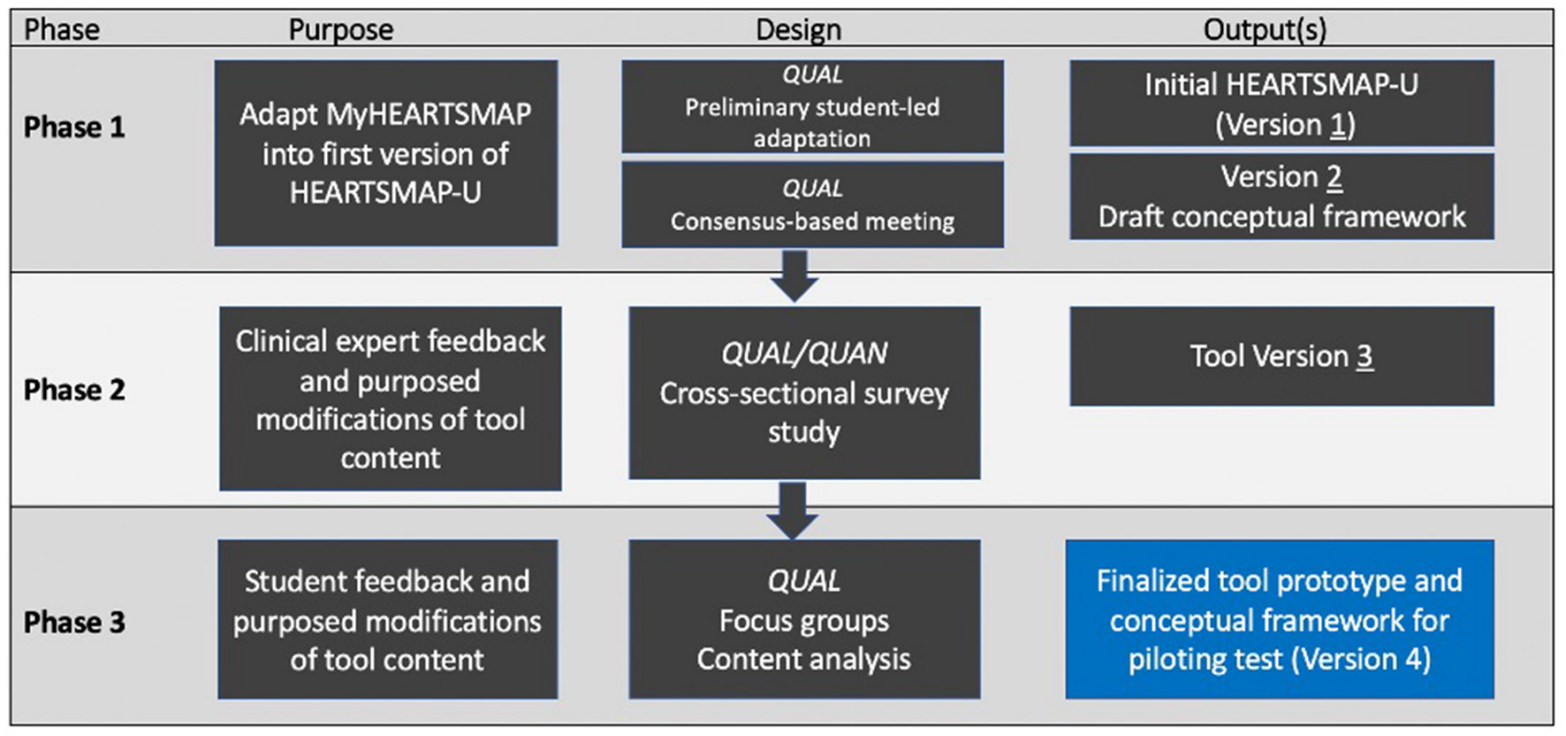
Schematic outlining our reported multiphasic tool adaptation process. Figure adapted from Riff et al. (59). QUAN, quantitative; QUAL-qualitative.

Our tool adaptation process includes three phases and has been informed by established guidelines for developing patient-reported outcome measures in the literature (55–58), and expertise from diverse stakeholders including clinical experts and student end-users. We used an iterative, multi-method approach, outlined in Figure 1. For each phase we describe the design, study procedures, and analytic approach. We obtained approval from our institutional research ethics board for Phase two and three, in which research participants were recruited.

### Phase One: Collaborative Working Meetings

#### Design

We conducted virtual working meetings between November 2018 and April 2019 with a diversely assembled study team of students and co-investigators. The purpose of our one-on-one student consultations was to generate ideas on how HEARTSMAP-U needs to be adapted for fitness for purpose in the university context, through a collaborative and consensus-based process. Our co-investigators included a family physician, clinical psychologist, a youth psychiatrist, addiction psychiatrist, patient-reported outcome measurement expert, and a graduate student researcher. The purpose of our co-investigator meetings was to formalize HEARTSMAP-U's intended use and conceptual framework. This included ensuring the tool assessed relevant psychosocial stressors (e.g., student-specific, age-related), and that its resource recommendations were accessible and match desired clinical flow (e.g., how/when specific supports should be accessed).

#### Study Procedure

Prior to co-investigator meetings, we had a group of gender and racially diverse research students (medical/undergrad/ graduate) review the pediatric MyHEARTSMAP tool and change language and content to be suitable for the post-secondary student population. We did not put restrictions or parameters on student researchers proposed modifications. This exercise resulted in the first HEARTSMAP-U version.

Co-investigators used the first HEARTSMAP-U version and existing HEARTSMAP conceptual framework as a starting point for tool modification. Discussions were free-flowing and open-ended, and investigators' feedback/suggestions were not constrained to the measurement model and conceptual framework of existing HEARTSMAP tools. We used a consensus-based decision-making process. Proposed tool changes required 100% investigator consensus. When we could not reach consensus, we held discussions until all investigators came to agreement. The lead investigator (PV) took comprehensive notes documenting all team decision-making and made approved tool modifications between each meeting. We held meetings until the team collectively felt a clinically and contextually relevant tool version had been reached.

#### Analysis

Throughout all meetings, we summarized and reported general impressions and key discussion points. We made necessary tool modifications between co-investigator team meetings.

### Phase Two: Clinical Expert Review

#### Design

We conducted a cross-sectional survey study with Canadian mental health clinicians who support post-secondary students, guided by an expert review methodology (60).

#### Study Recruitment

We recruited a convenience and snowball sample of participants through our professional networks, until data saturation was reached. Participation was self-paced and took place remotely, over our secure study website from July 2019 to September 2019.

#### Study Procedure

Participants watched a mandatory 3-min instructional video, explaining study procedures, the digital platform, and HEARTSMAP-U (purpose, structure). Second, we asked participants to reflect on their professional experience and formulate a fictional clinical vignette describing a student presenting to their practice in psychosocial distress (mild to severe). Clinicians were expected to provide a brief description of their vignette and used this information as they progressed throughout the tool.

Next, for each tool section, clinicians reviewed all HEARTSMAP-U guiding questions and scoring criteria, scored their fictional students' concerns (if any), and completed a survey item asking “*Do HEARTSMAP-U's [guiding questions/scoring descriptors] sufficiently capture the full range of [section]-related stressors that youth in your practice might experience? (yes/no)”* As a follow-up item, irrespective of their prior response, all participants were asked to provide a qualitative response to “*what could be added or changed so the [guiding questions/scoring descriptors] better capture the range of concerns students may experience in relation to [section]?”* Clinicians also provided high-level feedback (e.g., tool impressions, content suggestions). All qualitative responses were collected through open-ended survey questions (textbox response).

After scoring all sections, clinicians reviewed tool-generated support recommendations and assessed whether they over- or underestimated fictional students' needs. Clinicians had the choice of completing a second evaluation with a new vignette. Upon study completion, the core research team analyzed all feedback and found opportunities to further adapt each tool section (e.g., content, language), to ensure it covers a full range of concern severity, both in terms of distress and functional impairment. We used the HEARTSMAP-U version resulting from Phase two as a starting point for Phase three student focus group discussions.

#### Analysis

We summarized clinician demographics and responses to dichotomous survey items (yes/no) as counts and proportions. A blended/abductive approach to qualitative content analysis was taken to synthesize and analyze all qualitative responses (61). Based on an initial, holistic exploration of the raw data (inductive process) and existing healthcare measurement literature (deductive process) (62), we developed a tentative coding framework that would encompass participants' qualitative responses (e.g., content coverage, context of use, etc.). We coded qualitative data in three cycles, each introducing an added layer of interpretation and data abstraction. Our research team used reflective memos documented throughout the data collection stage to support the coding process and interpretation. First, we conducted attribute coding, whereby all qualitative survey feedback was structurally coded and organized by tool section, to support feedback interpretation. Second, we conducted descriptive coding and, for each tool section, mapped all clinician responses to our pre-defined coding framework/categories. We separately analyzed and coded guiding question and scoring descriptor feedback. Third, we performed pattern coding to explore variations and sub-categories within existing codes. For clinicians who responded “no” to whether guiding questions and/or scoring descriptors aligned with their professional experience, we coded their qualitative responses into the most appropriate feedback category. For each tool section and feedback category, we report count data on the total number of clinicians/responses that map to them. Two investigators conducted qualitative coding, HB (first cycle) and PV (second, third cycle). We conducted analyses using Microsoft Office Excel and NVivo 12.0.

### Phase Three: Student Focus Groups

#### Design

We conducted a qualitative study with UBC-Vancouver students, guided by cognitive testing and iterative design methodologies (63–65). Similar to Phase two, we incorporated a variation of verbal probing, asking participants targeted questions on tool content and functionality. Through a series of sequential focus groups, we iteratively modified HEARTSMAP-U based on participants' feedback on guiding questions, scoring criteria, tool language (e.g., unclear, insensitive), and other suggestions (e.g., new tool section, format/structure). Focus groups took place between November 2019 and May 2020. Initially, we held in-person sessions, but later made them virtual, to allow remote participation and compliance with COVID-19 restrictions.

#### Study Recruitment

We recruited students through an existing partnership with university administration, health centers, and student organizations. Prospective participants completed an online expression of interest and demographic form. Using this information, we recruited a purposive sample of UBC-Vancouver students ages 17 years and older and setup heterogenous focus groups. We strived for proportional representation of the overall UBC student population across demographics: age, gender and sexual identity, program-type, year of study, race/ethnicity, international/domestic status, and lived mental health experiences (Web-Appendix B). We excluded students uncomfortable with being audiotaped.

#### Study Procedures

During each focus group, we first supplied participants with a high-level introduction to HEARTSMAP-U (e.g., purpose, components). Next, we reviewed tool components (guiding questions, scoring criteria), for each tool section. During this time, we asked participants to share their first impressions and engage in a dialogue around the tool's (1) comprehensiveness (*issues important to you and your peers*). (2) Relevance (*realistic content reflecting your experiences)*. (3) Understandability (*easily understood language)*. We encouraged participants to suggest tool modifications for the study team's consideration, either through group discussion or written feedback. We audiotaped focus groups, had them professionally transcribed (verbatim), and compared them against the original audio to confirm accuracy.

#### Analysis

We conducted two sets of analyses using focus group data. Consistent with analytic guidelines, we treat the focus group as our unit of analysis (66). First, between each focus group, the core research team reviewed RA notes documenting tool modifications proposed by students. For each comment or suggested modification, we took into consideration the general response from other focus group members (e.g., endorsed, objected) and whether it was consistent with clinical guidelines and earlier focus groups. Focus groups were held until a point of data saturation was achieved, whereby no new feedback was received that investigators had not already considered or considerations were mostly minor (e.g., word choice, grammar) (66).

After reaching sufficient data saturation, we performed an in-depth, abductive qualitative content analysis, with inductive and deductive components, using verbatim transcripts and research memos. First, an investigator (RA) deductively conducted attribute-based coding, to organize and sort all student comments by session and tool section. A second investigator (PV) interpretatively performed descriptive coding using Stewart et al.'s framework to categorize sectional feedback as either content or format/interface-related (67). Tool content-related feedback and modification suggestions were further analyzed through pattern coding using two additional frameworks. The COSMIN content validity framework and it's operational definitions for content relevance, representativeness, and understandability were used to analyze and characterize students' proposed modifications with respect to these categories (55). Coons et al.'s framework was used to assess modifications as either (1) minor, those not expected to change content or meaning (e.g., switching format from paper to online). (2) Moderate, subtle content/meaning changes (e.g., item wording, ordering). (3) Substantial, extensive content/ meaning changes (e.g., changing response options, new guiding questions) (68). Inductive, descriptive coding was also performed to characterize and report comments and feedback that did not fit within our a priori analytic frameworks.

For each tool section, we report representative quotes for each modification-type and inductively derived category, and reference quotes by focus group number (*FG X)*. We summarize participant sociodemographics using descriptive statistics and conduct the Chi-square test of independence (alpha = 0.05) to compare the demographic profile of participating students with those who expressed interest but did not take part in the study (e.g., not invited, declined).

## Results

### Phase One: Collaborative Working Meetings

A total of five students took part in preliminary tool adaptation activities, two undergraduate students and three medical students. Subsequently, we had five co-investigators who took part in three rounds of discussion and iterative tool modification, at which time all co-investigators agreed on HEARTSMAP-U prototype content. One clinical investigator took part and contributed feedback outside of organized group discussions.

#### Conceptual Framework

**Figure 2:**
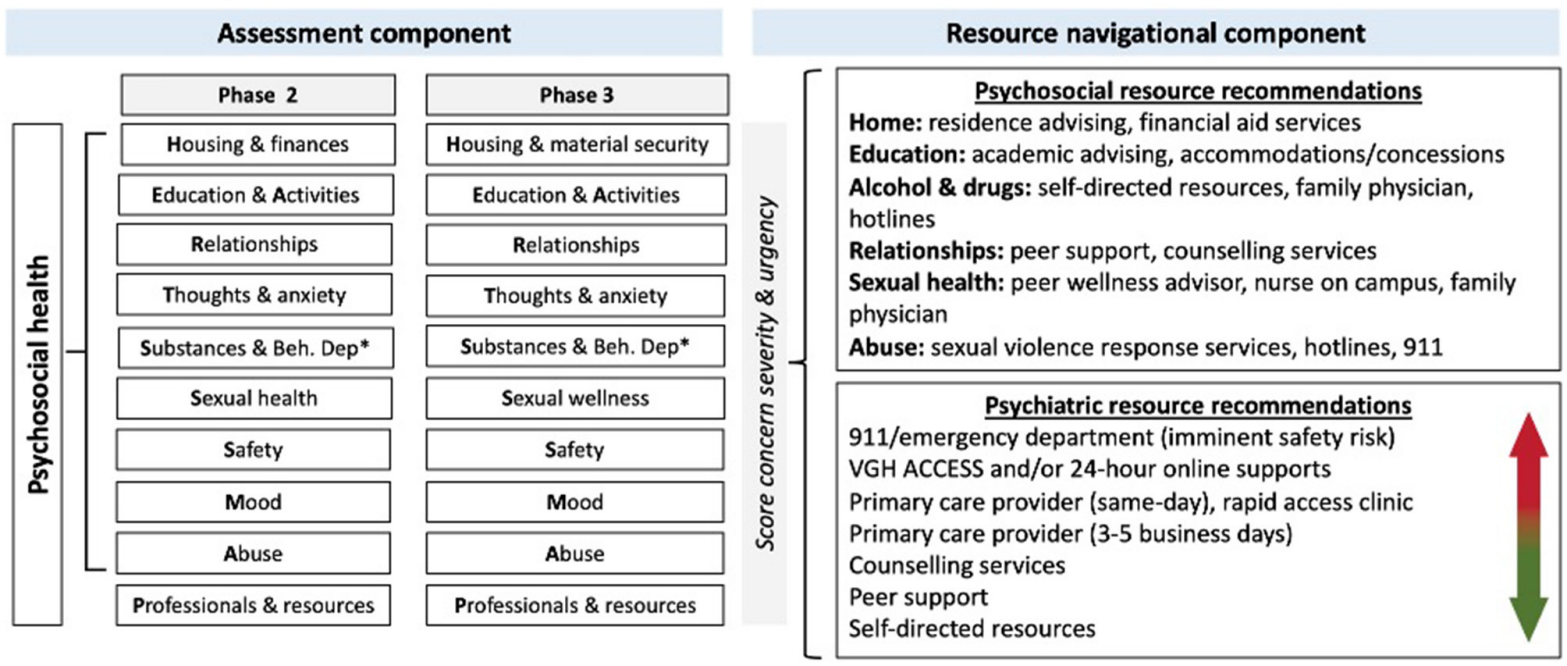
Conceptual framework of the finalized HEARTSMAP-U tool version, following adaptation among Canadian mental health professionals (Phase 2) and post-secondary students (Phase 3).

We largely retained MyHEARTSMAP's conceptual framework, recognizing universality of the measured constructs, however several sections were redefined. MyHEARTSMAP's “Home” section only measures the safety and supportiveness of the home environment, which may not encompass the transient nature of student housing. For HEARTSMAP-U, we modified this section into “Housing arrangements and finances” to include an assessment of housing stability and ease of managing housing-related responsibilities (e.g., paying bills, cleaning, cooking, etc.), in addition to housing safety/supportiveness. Finalized construct definitions are reported in Web-Appendix C and our conceptual framework is illustrated in Figure 2.

#### Tool Content and Resource Recommendations

Investigators decided MyHEARTSMAP's severity scoring spectrum (none to severe) required modification to accurately reflect the student population. “Alcohol and drugs” needed to reflect the social acceptability of leisurely drinking and marijuana usage among young adults. For several sections, investigators agreed that two different concepts were being measured together (e.g., thought disturbances and anxiety) which needed to be consistently assessed and delineated across all severity levels using “OR” Boolean operators. The team modified HEARTSMAP-U's resource recommendations so that they reflected the appropriate tier of resources/services as outlined by the post-secondary institution (69). Investigators identified opportunities to incorporate strength-building recommendations, triggered when students report no more than mild concerns. Feedback across all three working group sessions is summarized by tool section in Web-Appendix C.

### Phase Two: Clinical Expert Study

Participating mental health clinicians (*N* = 28) mostly identified as women (89%) and worked at large-size Canadian post-secondary institutions (96%). Most clinicians were either registered counselors (32.1%) or psychologists (32.1%) and affiliated with their institutions counseling (60.9%) and/or health services (30.4%). Complete demographic details are summarized in Table 1.

**Table 1:** Demographic characteristics of phase two participating clinicians.

**Characteristics**	***N***_total_ **= 28 (%)**
**Gender, Female**	25 (89.3%)
**Provider type**	
Psychologist	9 (32.1%)
Registered counselor	9 (32.1%)
Social workers	6 (21.4%)
Mental health nurse	2 (7.1%)
Family physician	2 (7.1%)
**Campus provider, yes**	28 (100)
**Affiliated services** ^a^	
Counseling service	28 (60.9)
Student health services	14 (30.4)
More than one	4 (8.7)
**Institution size** ^b^	
Medium	2 (7.1%)
Large	26 (92.9%)

a *Total proportion exceeds 100% as several clinicians held multiple affiliations*.

b *Large-size institutions were defined as those with a student population larger than 30,000, mid-size institutions were defined as having a student population between 10,000 and 30,000 students*.

#### Fictional Vignettes

Clinician-prepared vignettes scored across severity levels (0–3) for all sections, except “Alcohol and drugs” and “Abuse” which were only assessed on no (0) to moderate concerns (2). Of the 46 completed fictional cases, most described mild (46%) or moderate (44%) psychosocial concerns. Of cases reporting psychological challenges, participants assessed 20% as being severe, compared to only 2–4% of cases reporting on other psychosocial issue. A total of 18 (64%) clinicians decided to complete a second vignette evaluation and 17 (61%) expressed interest in referring a colleague to join the study.

#### Section-Level Review

Participating clinicians felt that HEARTSMAP-U's guiding questions (46–86%) and scoring criteria (54–82%) aligned with their own clinical characterization of each tool section. A majority felt the tool was “*very thorough,”* guiding questions were “*simple yet broad”* and scoring criteria were “*easy”* to understand and there was “*nothing to add.”* Conversely, 14% (Housing; Professionals and resources) to 54% (Education and activities) and 18% (Housing; Abuse) to 46% (Sexual Health) of clinicians felt that HEARTSMAP-U's guiding questions and scoring descriptors, respectively, required more characterization to match their observations of each psychosocial construct. From clinicians' qualitative responses, we derived four categories that feedback was related to: (1) coverage of concern severity, consistent with the tool's intended use; (2) tool suitability in the clinician's own context-of-use; (3) minor language/wording issues with minimal impact on sectional content/meaning; and (4) content that clinicians perceived as missing but was elsewhere in the tool. We elaborate on each of these themes below. Counts and proportions summarizing participants' feedback by coding category are summarized in Tables 2, 3, for guiding questions and scoring descriptors, respectively.

**Table 2:** A breakdown of phase two clinician's feedback on HEARTSMAP-U's guiding questions.

**Total number of clinicians** (***N*****_Total_ = 28**)	**Guiding question alignment^a^**		**Clinician count per feedback category^b^**				
**Tool section**	**Yes**	**No**	**Concept coverage**	**Context of use**	**Language**	**Covered elsewhere**	**Unclear**
Housing	24	4	1	3	0	0	0
Education and activities	13	15	3	6	0	6	0
Alcohol and drugs	18	10	2	7	0	1	0
Relationships	17	11	4	5	0	2	0
Thoughts and anxiety	17	11	2	4	0	5	0
Safety	16	12	4	0	1	6	1
Sexual health	14	14	0	7	1	6	0
Mood	14	14	5	3	1	5	0
Abuse	16	12	3	7	0	2	0
Professional and resource	24	4	1	2	1	0	0
**Total^c^**		**107**	**25**	**44**	**4**	**33**	**1**
			**23%**	**41%**	**4%**	**31%**	**1%**

*Each count represents a unique clinician. Under each “feedback category”, counts represent the number of unique clinician's whose qualitative response mapped to that respective category. Each clinician's qualitative response mapped to a single feedback category, based on the focus of their concerns*.

a *Clinician response to whether guiding questions captures the full range of section-related concerns seen in their own practice (yes/no)*.

b *Number of clinicians who felt guiding questions did not align with their professional experience (responded “no“), stratified by the feedback category most closely relating to their comments/suggestions*.

c *Total counts and percentages of qualitative responses (i.e., clinicians) per feedback category*.

**Table 3:** A breakdown of phase two clinician's feedback on HEARTSMAP-U's scoring descriptors.

**Total number of clinicians** (***N***_Total_ **= 28**)	**Scoring descriptor alignment^a^**		**Clinician count per feedback category^b^**				
**Tool section**	**Yes**	**No**	**Concept coverage**	**Context of use**	**Language**	**Covered elsewhere**	**Unclear**
Housing	23	5	2	0	0	3	0
Education and activities	16	12	3	4	0	5	0
Alcohol and drugs	19	9	5	4	1	0	0
Relationships	22	6	3	2	0	1	0
Thoughts and anxiety	22	6	1	2	1	2	0
Safety	20	8	2	3	2	3	0
Sexual health	15	13	2	5	0	6	0
Mood	18	10	6	2	0	0	2
Abuse	23	5	2	1	0	2	0
Professional and resource	19	9	6	0	3	0	0
Total^c^		**83**	**32**	**23**	**7**	**22**	**2**
			**39%**	**28%**	**8%**	**27%**	**2%**

*Each count represents a unique clinician. Under each 'feedback category', counts represent the number of unique clinician's whose qualitative response mapped to that respective category. Each clinician's qualitative response mapped to a single feedback category, based on the focus of their concerns*.

a *Clinician response to whether scoring descriptors captures the full range of section-related concerns seen in their own practice (yes/no)*.

b *Number of clinicians who felt scoring descriptors did not align with their professional experience (responded “no”), stratified by the feedback category most closely relating to their comments/suggestions*.

c *Total counts and percentages of qualitative responses (i.e., clinicians) per feedback category*.

##### Sectional Content Coverage

Respectively, 23 and 39% of all guiding question and scoring criteria comments focused on how well sections captured behaviors and experiences necessary for students to be able to self-evaluate the presence of concerns, across the entire spectrum of severity. Two major sub-categories emerged from these comments: improving scale gradation and broadening content. Clinicians felt scoring descriptors needed to accommodate students who may fit “*in-between”* existing criteria. For example, in the “Relationships” section, one participating clinician suggested we:

“*Address [the] situation where someone is not losing connections but is working onbuilding confidence to have romantic connections.”*

We changed the score 1 descriptor to include instances where students may have emotionally supportive connections but may struggle to build or maintain them. For “Education and activities,” clinicians indicated two instances where partial criteria could be met, and students may struggle to score themselves:

“*Need to capture that mental health concerns are impacting academic performance, but student is still actively engaging in studies”*

“*Need options that capture languishing in one area only. Academics and activities are separate constructs. You can be functioning in one and not in the other.”*

We used an “OR” Boolean operator to create two scoring pathways across scores 1–3, distinguishing academics from other activities, and allowing students to select the most severe score applicable to their situation. Under score 1, we have also taken into consideration instances where students may be engaged in class, but their academic performance may be declining.

Feedback often focused on broadening certain criteria and guiding questions to encompass a larger cross-section of the general student population, examples include:

“*What about behavioural addictions (e.g., gambling, gaming)”* (Alcohol and drugs)

“*Include family relationships”* (Relationships)

“*Financial abuse is not listed - some family members have taken a client's student loanand used it for themselves”* (Abuse)

“*Needs to encompass more range of emotions - anger and shame in particular aremissing.”* (Mood)

The “Alcohol and drugs” section was expanded to include additional substances (marijuana, prescribed medication, illicit substances) and behavioral addictions (e.g., excessive exercise or sex, gambling).

##### Context-of-Use

A sizable proportion of guiding questions (41%) and scoring criteria feedback (28%) focused on introducing a diagnostic level of detail and specificity to each section's content. Clinicians requested the tool assess sub-categories of its existing broad psychosocial areas. For example, in the “Relationships” section, clinicians felt that HEARTSMAP-U did not explore specific relationship types or problems and they proposed guiding questions that consider:

“*Parental expectations to perform or excel impacting relationships, being able tocommunicate with one's parents.”*

“*Break-ups specifically”*

“*Friends nearby, versus those only met through social media (and not physicallyavailable)”*

HEARTSMAP-U's general assessment of relationship challenges at mild, moderate, and severe levels is suitable for its intended use, as a multi-domain psychosocial screen. Feedback calling for added detail and subcategorization were deemed by the study team as most relevant to the clinician's own assessment context, rather than initial screening purposes. A lengthier tool may also reduce usability and increase respondent burden.

##### Content Covered Elsewhere and Language

A large portion of concerns raised with guiding question (31%) and scoring descriptors (27%) had already been addressed in different tool sections, that participants may not have yet reviewed. In the “Thoughts and anxiety” section, participants expressed that:

“*I'm not sure if this is coming later in the questionnaire but adding more depressive symptom questions. Perhaps that will be in the mood section I haven't come to yet.”*

Language-related concerns made up a small proportion of guiding questions (4%) and scoring descriptor feedback (8%). These comments flagged language that students may misinterpret or find confusing such as “*psychosocial,” “intoxication,”* and “*intrusive thoughts.”* In another instance, participants felt the tool's singular use of “*partner,”* may stigmatize students in polyamorous relationships.

#### Resource Recommendations

Participants rated the appropriateness of 265 triggered recommendations and perceived that most recommendations (70%) were consistent with the fictional students' support needs. A smaller portion of participants felt tool-generated recommendations underestimated (18%) or overestimated (12%) support needs. Participants also expressed concerns with recommending emergency services (e.g., 911, emergency department) in the absence of imminent safety risk. Rather, participants considered same-day primary care, rapid access clinics, and 24/7 e-counseling as appropriate supports.

### Phase Three: Student Focus Groups

Demographic distributions did not significantly differ (*P* > 0.05) between participating students and those not invited to a focus group session (non-participating students). A total of 54 students took part in 6 focus group sessions, each 2 hours in length. We had nearly equal proportions of student's aged 18–21 (50%) and 22–25 years (48.1%). Approximately two-thirds of participants identified as female and undergraduate students. Most participants were in their first or second year (61.1%), living off-campus (57.4%), identified as straight (72.2%) and as part of a visible ethnic minority (53.7%). Over 80% reported experiencing mental health challenges in the past (72.2%) and/or present (55.6%). Demographic details are summarized in Table 4.

**Table 4:** Demographic characteristics of phase three student participants and non-participating students who expressed interest.

***Characteristics***	**Study participants (*N* = 54)**	**Non-Participants (*N* = 152)**
	***N* (%)**	***N* (%)**
**Age (years)**		
18–21	27 (50.0)	89 (58.6)
22–25	26 (48.1)	52 (34.2)
26 and older	1 (1.85)	11 (7.2)
**Program of study**		
Undergraduate	36 (66.7)	116 (76.3)
Graduate	9 (16.7)	19 (12.5)
Professional program	9 (16.7)	17 (11.2)
**Year of study**		
1 and 2	33 (61.1)	77 (50.7)
3 and 4	16 (29.6)	59 (38.8)
5 +	5 (9.26)	14 (9.2)
Living arrangements, on-campus	23 (42.6)	65 (42.8)
**Ethnicity**		
Visible ethnic minority	29 (53.7)	73 (48.0)
Aboriginal Person	1 (1.9)	4 (2.6)
Caucasian	24 (44.4)	75 (49.3)
**Gender identity**		
Female	35 (64.8)	117 (77.0)
Male	19 (35.2)	31 (20.4)
Non-binary	0 (0.0)	4 (2.6)
**Sexual Orientation**		
Straight	39 (72.2)	118 (77.6)
Queer/questioning	15 (27.8)	34 (22.4)
**Type of student**		
International student	18 (33.3)	45 (29.6)
Domestic student	36 (66.7)	107 (70.4)
**Mental health concerns^a^**		
Past	39 (72.2)	105 (69.1)
Present	30 (55.6)	88 (57.9)

a *Total proportion exceeds 100% as participants could check-off multiple options*.

Earlier focus groups emphasized substantial content-related modifications (*FG 1-3*) relating to relevance and representativeness of HEARTSMAP-U's content. In later sessions, students raised mild/moderate content suggestions. The proportion of focus group participants engaged in group discussion remained consistent across sessions (Web-Appendix D).

#### Interface Modifications

Participants suggested multiple interface-related modifications summarized below. First, a privacy disclaimer at the beginning of the tool, so the user is aware of the scope, intended purpose, and confidentiality implications associated with completing HEARTSMAP-U. Second, a progress bar with a coordinated color scheme (e.g., green complete, orange=in-progress), to motivate users in completing the tool. Third, users should have the ability to download screening results to potentially share with their care provider, and that tool recommendations link to service information the user can directly act on. Finally, participants felt that pairing the tool with calendar apps would ease repeat screening and booking appointments.

#### Content Modifications

##### Representativeness-Related

When probed, students did not name any novel psychosocial concepts that were completely missing from the tool. However, in session one and two, participants felt that a student's financial situation is a crucial stressor that contributes to their mental well-being, however its assessment in the tool was limited to housing-related finances (e.g., bills, rent). One participant summarized the issue as:

“*Regarding finances, this is quite broad, perhaps distinguish between living affordability (house/shelter, food, health) and school (tuition); perhaps a better term would be security or financial stability/security.” (FG1)*

After the first focus group session, study investigators revised the overall concept to measure ‘Housing and Material Security,' shifting the focus away from strictly housing and financial difficulties and assessing whether necessities in general were met or not. Figure 2 displays our conceptual framework prior to and following focus groups.

##### Relevance-Related

Across all focus group sessions, most students felt that HEARTSMAP-U's psychosocial areas applied to their lived experiences and captured the challenges they experience within and outside the post-secondary educational context. One student described the tool's multi-dimensional nature as:

“*Going into the different facets could be really helpful… people sometimes underestimate how much other stuff can really influence their mental health. Like if you're reallystruggling with school or rent money, that really has an impact on mental health. Butsometimes we don't realize it. We just think oh, it's because I'm just having a hardtime.” (FG 4)*

Participants found the graded scoring spectrum to be an important attribute as it recognizes a middle ground, which could allow more students to see themselves in the options. One participant expressed:

“*I like the use of “but” in [the] sections, a lot of questionnaires have all or nothing questions when sometimes you do struggle with the problems but have implemented coping skills.” (FG 3)*

However, many students expressed concerns that the scoring gradation was not always clearly delineated in psychiatric sections such as “Mood” and “Thoughts and Anxiety,” as participants felt that descriptors for scores 0 and 1 were “*blurred”* and they “*had a little bit of trouble distinguishing them.”* Students also felt that descriptors should emphasize functional impairment and “*refer more to actions”* associated with various levels of concern severity, as opposed to just focusing on how students are feeling. Participants also found score 0 to be strength oriented whereas the remaining options reflected a gradient of deficits. They felt the score 0 language should be more neutral, and unassuming that the student is flourishing. One participant suggested:

“*Resolve the language for 0 since it seems— it sounds a little idealistic for students. Instead of the word 'satisfied', …say like, “I'm keeping up and maintaining my academics and activities”…I think that would be a better capture of the baseline.” (FG 2)*

Where applicable, the research team changed the scoring criteria to have a more consistent pattern across sections. A score 0 would indicate no perceived challenges (neutral), a 1 would indicate challenges with no to minimal functional impairment or distress (i.e., can still go about self-care/daily activities), a 2 would indicate challenges with moderate functional impact (i.e., difficulties going about self-care/daily activities), and a 3 representing challenges with severe impairment/distress, preventing self-care and daily activities. Participants in later sessions affirmed and supported these changes.

For each section, students highlighted opportunities to refine content and improve its relevance in assessing the concept it maps to. For example, in session one and two, participants expressed that engagement level and satisfaction should be included in “Education and Activities” to help evaluate how academics and extracurricular activities interact with students' mental well-being. One student expressed that being “*engaged and [also] unsatisfied should be included [in the tool] because in that way [the University] can measure how meaningful or successful the activities [on] campus are for students.” (FG 6)*. In another instance, participants felt the “Mood” section overly focused on sad or anger-related emotions and needed to incorporate situations where students may perceive “*no emotions or numb.”* For “Sexual wellness,” students felt that score 0 (healthy sexual relationships) needed to clearly reflect protection-less, consensual sex between long-term, responsible intimate partners, and score 3 (high-risk behavior) needed to integrate discussion of capacity to consent. Table 5 reports representative quotes and corresponding modifications relating to student's perception of HEARTSMAP-U's relevance.

**Table 5:** Content-relevance related phase three student feedback with representative quotes and tool modifications.

**Tool section**	**Key feedback**	**Representative quotes**	**Tool modifications**
**Housing and material security**	Take into consideration that while needs are met, the student may not be satisfied with how well or easily they are being met.	“*If I can't pay for my housing, my parents are always… going to have my back…But my finances are a completely different situation…like, not being able to go out with friends because I'm just thinking about the future and, oh man, I'm going to have to put money towards this and that.…[this describes] a lot of I think first year [students]…especially people living on campus.” (FG 3)*	Scoring criteria: assess how easily and satisfactorily students perceive their needs being met.
	Expand on what falls under finances and material needs	“*Housing, food, rent, tuition, those are the only four you're interested in? Are there additional ones that we're supposed to know are material needs? I just don't know if those are just examples and there's many, or if those are just, like, specifically those four.” (FG 4)*	Term definitions: add “housing, food, rent, tuition, insurance, medication” as examples of needs
**Education and activities**	High GPA does not mean a student is engaged with or enjoying what they are studying	“*I feel like this has a lot to do with motivation, like whether you like or not. I mean, I did do well. I wouldn't say I was struggling, it's just I didn't feel motivated to do it, but I was doing it either ways. Because I need my GPA but I wasn't happy.” (FG 1)*	Guiding questions: add-in “Do I feel motivated to engage in my academics and activities?”
	Motivation may not be the best word choice for this section.	“*In my experience, my friend's experience, a lot of it is just life gets overwhelming and it's not like… you've lost motivation in your schoolwork. It's that there's just so many other things going on that your academics start slipping...It's not because you don't want it to...Maybe you have, like, a breakup going on or maybe you're moving… there's just a lot of other factors…grouping it under lost motivation and having it underlined and bolded, doesn't really do justice.” (FG 3)*	Scoring criteria: captures engagement and satisfaction with engagement, rather than strictly motivation.
	Resolve language to reflect that the student is unable to engage in their academics, not by choice.	“*Instead of saying, I have completely stopped engaging with academics or activities, etc., I would more lean towards the side of, like, I have been unable to. Yeah, because it's not really like the student choosing to completely stop, right. It's like them not being able to anymore.” (FG 5)*	Scoring criteria: change “I have completely stopped” to “I have been unable to” engage with…
**Relationships**	Clarify how relationships is being defined and assessed.	”* Relationships are often thought of as more intimate, and I think that changing [relationships] to 'social connections' would work well.“ (FG 6)*	Word choice: change relationships” to “social connections”
		“*For zero it says, I am emotionally supported and satisfied with my social connections. But for one, two and three it focuses on the word “relationships.” …Social connections seem more broad, whereas relationships seems like they are referring to something more personal… If the goal is to be more broad, maybe social connections would reach a wider audience.” (FG 6)*	
**Thoughts and anxiety**	Frequency and time frame can make it easier for students to place themselves on a score.	“*Frequency is a good measure of the intensity of someone who has anxiety disorder. I don't have anxiety disorder, but I think it'd be really helpful if someone the person...getting their assessments, and if they feel comfortable filling that out [could] differentiate between their intensity.” (FG 2)*	Scoring criteria: incorporate frequency descriptors (e.g., sometimes, often)
	Language can be resolved to sound less accusatory.	“*The language of losing control feels a little accusatory. If it could be more like you feel out of control. That way that things like clearly an emotion you are in control of and not something you're doing wrong.” (FG 5)*	Scoring criteria: “I am losing control” changed to “I feel like I am losing control
**Substances and behavioral dependencies**	Recreational substance use and addictive substance use should be differentiated.	“*Especially in a university culture, there's a difference between – abusing drugs, and recreationally using it, and addiction. If you just want to focus on the dependence and addictive behaviors, I think it'd be good to clarify or specify that these drugs – or some drugs are getting in the way of my life (FG 2)*	Scoring criteria: incorporates concepts of dependence and levels of functional impairment (e.g., disruption to daily activities/self-care)
	University “norms” may not be healthy and can be excessive.	“*I would kind of hesitate against using norms because I think in university it can be a norm to kind of drink quite excessively. And that is still problematic in and of itself.” (FG 4)*	Term definitions: “norms” was removed.
	Non-suicidal self-harm may be more severe than how it is currently recognized.	“*People who would put themselves in number one, [could also] end up in number three, like they have a suicide plan, but maybe they're self-harming as well...So then that might just create a discrepancy.” (FG 4)*	Clarification: users score the most conservative/severe scoring options that applies to them.
**Safety**	Non-suicidal self-harm can be therapeutic, however past history is strong predictor of suicidal behavior.	“*Non-suicidal self-harm is actually a really therapeutic coping mechanism to the patient. Because it's kind of like their way of dealing with it, and if you take it away, then they kind of may progress to doing worse things. So, I see why it is a one… Add like past history of attempted suicide [to score three]. Because I know that's a huge risk for future suicide.” (FG 5)*	Scoring criteria: maintain non-suicidal self-harm as score 1 and add “previous suicide attempts” to score 3.
**Sexual wellness**	Include scenario where one partner may not use protection.	“*Maybe under one, you could say, ‘I always use protection but I'm unsure or I know that my partner doesn't use one.”' (FG 2)*	Scoring criteria: incorporate uncertainty around partner's sexual wellness or risk-taking.
**Mood**	Clarify that changes in daily activities/self-care are in relation to mood changes.	“*[In score 3] I'd be good to specify because of elevated or low moods…I feel like you can have sleep, energy, diet changes that prevents you from going about your day for at least a week if you're stressed.” (FG 2)*	Scoring criteria: clarify connection between functional impact and mood.
	Include perceived numbness/lack of emotion.	“*The flat affect that some people can get when they're depressed is not really being captured all the time in any of these kinds of four questions…Because they might not feel down, but just the things that make them happy, no longer make them happy.” (FG 4)*	Scoring criteria and guiding questions: incorporate numbness and flat affect.
**Abuse**	Section will prevent students from slipping through the cracks.	“*I think what's important about this tool is that someone who would be saying that they believe that this is happening to them and scoring a one, two or three would hopefully then get the resources where they'd be able to talk about it further or more… Not have people slip through the cracks of the questionnaire. So I think that's great.” (FG 6)*	No substantial modifications were made.
**Professionals and resources**	Consider the situation where some but not all needs are met.	“*I am supported in some of my mental health needs but need further support.” (FG 1)*	Scoring criteria: recognize partial resource connection into score 1.
	Commenting on helpfulness of existing care	“*I like the inclusion of 'I didn't find it helpful'.” (FG 5)*	No modifications made.

##### Understandability-Related

Students agreed that overall, HEARTSMAP-U's scoring descriptors, guiding questions, and purpose were clear and easily understood. Guiding questions were perceived helpful and provided additional “*clarification”* on the section to be scored. Students suggested multiple modifications to improve content understandability. In session one, participants felt many terms and phrases (e.g., control over thoughts, basic needs, emotional support) were unfamiliar or ambiguous. Participants expressed the need for a “*common language”* between the tool user and researcher, so students comprehend questions and scoring criteria as intended, “*that way connotations aren't playing as much of a role.”* In response to this, we introduced a ‘hover-over' feature for any term or phrase students expressed uncertainty or confusion. In sessions 2-6, students consistently expressed approval of this feature and built a library of concise definitions with student-friendly language. Students stressed the need for a clear instructions page at the beginning of the tool, to ensure students knew how to approach each section. Participants felt if the user is uncertain between two scoring options (e.g., score 1 or 2), they should select the most conservative/higher score that applies to their situation. For example, under “Relationship,” “*some relationships might be fine, but others aren't. Then you're basing [your score] on the struggling ones.”* Table 6 summarizes participants comprehension-related feedback for each tool section, followed by the study teams agreed upon modifications. Overall, we found students in later sessions (5-6) affirmed and supported content modifications made in response to concerns raised in earlier sessions (1-4).

**Table 6:** Comprehension-related phase three student feedback with representative quotes and tool modification.

**Tool section**	**Key feedback**	**Representative quotes**	**Tool modifications**
**Housing and material security**	Unclear whether relational stressors are relevant here.	“*With the pandemic, I've read many articles that say that a lot of students that are a part of the LGBTQ community…have to go back home. That is not considered, like, a safe space for them because their parents reject them, or they suffer domestic violence… I thought [this section] meant a little bit more of that.” (FG 6)*	Scoring criteria: assess how easily and satisfactorily students perceive their needs being met.
		“*When you say safe and secure, does that also include, like a stressed home kind of thing? Because I know sometimes at home, yeah, you can feel safe, but it can be very stressed from time to time.” (FG 4)*	Term definitions: added “housing, food, rent, tuition, insurance, medication” as examples of stressors associated with material security
	Appropriate to assessing needs.	“*Language is pretty good, and the categories are clear. Since this is meant to be a broad scanning, I do not think more information should be asked.” (FG 6)*	No modifications
**Education and activities**	Idealistic language should be avoided.	“*Language for [score zero]... sounds a little idealistic for students,... instead of the word ‘satisfied', we could maybe say like, “I'm keeping up and maintaining with my academics and activities'... a better capture of the baseline.” (FG 1)*	Word choice: changed to reflect a neutral perception toward one's academic situation.
	Normal versus overwhelming academic stress	“*I'm thinking that maybe there needs to be some clarification between just a normal level of overwhelmed with university work and feeling so overwhelmed you're paralyzed or whatever. Some differentiation between when it becomes mental illness and what's just normal levels of a lot of stress.” (FG 5)*	Point of clarification: feeling “paralyzed” is meant to be captured through functional impairment in relation to academics and extracurriculars.
**Relationships**	Wording in this section needs clarity.	“*I had an initial confusion in reading the first one. The, “I have emotionally supportive connections, but they're hard to build, maintain, or sometimes cause conflict.” I think my initial confusion was that it didn't make sense to me at the beginning...How can you have emotional supporting connections if they're hard to maintain, or if they're hard to build in the first place, or if they cause conflict? I feel like the language can be changed there.“ (FG 2)*	Word choice: changed throughout the entire section to reflect those students who may feel supported but still struggle with relationships. Ex. Score 1 changed to “I feel emotionally supported but feel challenged building/maintaining social connections.”
	Good use of “overwhelm”	“*I like overwhelmed. I actually really like the fact that you kind of have two dimensions where it's, like, whether or not you're feeling supported and kind of capturing whether or not you're actually able to kind of maintain those relationships. Because sometimes you can have a very good support network but just feel so overwhelmed with things, that you don't feel like you can access it or it's really hard to kind of maintain that. And I really, really like… and I have never seen anybody kind of ask it in that way.“ (FG 4)*	No modifications
	“Overwhelmed” it can be positive or negative	“*Overwhelmed could be, like, positive, but it could also be negative. So in this case it would be negative I assume. Maybe specify, like, what exactly is overwhelming and again, defining building and maintaining relationships might be helpful.” (FG 4)*	Added hover over: for overwhelmed. Wording of this section was changed and overwhelmed was removed from scoring and added to a hover over.
**Thoughts and anxiety**	Clarify score 1 and self-care activities.	“*I think that number one needs some clarification. I feel like I sometimes lose control of my thoughts, but I can go about self-care daily activities. Do you mean that even though you sometimes lose control of your thoughts, with self-care activities, you can handle them? Or what do you mean?” (FG 6)*	Word choice: changed “I can go about“ to “I can keep up with” self-care/daily activities. Removed “always” from “always in control of my thoughts” (absolute language/unrealistic).
**Substances and behavioral dependencies**	Provide dependency examples	“*I think the more examples and the more definitions, it makes it more... accessible to people, by... explaining it in many ways as possible for people to identify and try to find where they want to scale themselves.” (FG 2)*	Additional examples: added “excessive sex/gambling/gaming/ exercise/eating/spending” as dependency examples to the hover-over.
**Safety**	Safety alert could be more supportive.	“*Saying ‘you need to connect to the crisis line' may sound a bit scary? Would people feel reluctant to do so?” (FG 6)*	Word choice: changed to “Immediate help is available. Click here to connect with a crisis responder now” (hyperlinked text).
**Sexual wellness**	Clarify methods of protection	“*Maybe specify what is meant by protection. Is it just physical, the condom, or birth control pills?” (FG 2)*	Additional examples: added “condoms, dental dams, contraception” to the hover-over.
	Consent should be discussed more	“*I noticed that consent was in the description for healthy sexual decisions. But I feel like it could also be more visible, because it's also… consent is a big part of sexual wellness.” (FG 6)*	Scoring criteria: consent incorporated into score 3: “at least one of us does not have the capacity to consent.”
**Mood**	Clarify mood changes	“*For Score 3, could it be changed to say ”have been swinging...“?” (FG 6)*	Word choice: instead of saying “mood swings”, change to “ swinging between the two extreme” low/numb and elevated/elated.
**Abuse**	Recognize effective coping with past abuse.	“*[change to] working through [past abuse] effectively instead of able to work.” (FG 3)*	Word choice: added “effectively” to score 1 and added a hover over with clarification.
**Professionals and resources**	Avoid absolute language	“*Say ‘satisfied” instead of supported in all' mental health resources (FG 1)*	Word choice: changed “supported with all” to “satisfied” with mental health needs.

## Discussion

We document the multiphasic adaptation of previously developed pediatric psychosocial assessment tools into HEARTSMAP-U, a version fit-for-purpose for the post-secondary student population. In Phase one, the study team arrived at a prototype considered clinically and contextually suitable for post-secondary students. In Phase two, participants saw alignment between HEARTSMAP-U's content and their clinical experiences. Of those who offered constructive feedback, most called for a diagnostic level of content detail and specificity (28–41%), which may not be relevant for screening purposes. Between 23 and 39% clinicians provided modifications/feedback related to sectional content and severity coverage, as per the tool's intended use as a self-administered screener. In Phase three, students provided feedback for improving the content relevance and understandability. Modifications focused on creating a common language between tool users and researchers, as well as ensuring scoring options were realistic and distinguishable. Students did not propose novel psychosocial domains that HEARTSMAP-U does not already directly or indirectly measure.

Our tool adaptation process and methods built on existing screening literature and prior student-specific, rapid screening tools described in the literature. The Symptoms and Assets Screening Scale is a lengthier (34-item) instrument, and its content focuses mostly on psychological concerns. In the absence of more generalized psychosocial screening, students' resource needs may be underestimated or only partially understood. The College Health Questionnaire addresses these concerns and allows for multiple-domain screening. Both previous instruments display promising reliability and construct validity evidence. However, reporting of their development process is limited and describes a traditional “top–down” approach, with little mention of student and/or clinical expert (non-investigators) involvement. Engaging the target population has important implications in refining tool content, language, and instructions, which would contribute evidence toward the instrument's content validity, helping to ensure the measure reflects students' lived experiences and vernacular (63). While not intended for screening or assessing mental health issues, the Post-Secondary Stressor Index (PSSI) is an institution-facing tool that evaluates students' exposure to stress and supports targeted mental health intervention/programming (70, 71). In developing and evaluating the PSSI through an extensive process of student engagement, Linden et al. noted that their tool saw markedly stronger psychometric properties compared to similar tool's previously developed without involving students. The ISPOR Patient-Reported Outcomes Good Research Practices Task Force Report highlights the critical contribution that end-user engagement makes to the content validity argument of an instrument and its quantitative psychometric properties (72, 73). In line with this literature, HEARTSMAP-U's adaptation was closely informed by students, content experts of their own lived experiences and the collective experience of being a post-secondary student. While previously described measures have focused exclusively on assessment, scoring on HEARTSMAP-U feeds into a complex decision-making algorithm to generate severity and urgency-specific recommendations for both psychiatric and social/functional resources. The tool's action-oriented approach to assessment may help avoid “run-around” and potentially unnecessary referrals to already scarce psychiatric services.

During Phase two expert review, a fraction of clinician's (23–39%) identified opportunities to improve severity coverage across HEARTSMAP-U's sections, particularly of concerns that fit in-between “none” and “mild” scoring options. Capturing subthreshold and milder cases is a critical challenge with existing self-report measures (74). If transient and non-severe issues are not explicitly reflected in the scoring criteria, these cases may go underreported due to stigma and remain unmanaged until crisis situations. Recently, transdiagnostic clinical staging models of mental illness have received great attention as an improved means of characterizing the progression of mental disorders into adulthood. HEARTSMAP-U's symptomatic and functional characterization of low to high severity concerns may support its screening utility for mental disorders at their earliest stages, from non-specific to subthreshold symptoms (75). A sizable proportion of clinician's (28–41%) provided feedback more suited for their own practice and context of use (e.g., diagnostic-level probing), which would not be consistent with HEARTSMAP-U's intended use as a brief screener. These comments may reflect outstanding assessment needs and challenges in the post-secondary counseling settings, where validated, standardized intake procedures/measures are infrequently used, difficult to interpret, and can be time-consuming (76). A number of clinician's (18%) scored the tool's resource recommendations as underestimating the support needs of their fictional case. This may have been an artificial finding reflecting our online survey setup, where we asked participants to assess the appropriateness of each individual recommendation. By design, HEARTSMAP-U pairs intensive and lower tier resources, recognizing that multiple treatment and self-management modalities can help students cope with the long-wait times associated with scarcely available psychiatric resources (77). We believe that if clinicians had been asked to holistically assess the appropriateness of their case's service recommendations all-together (low tier and intensive options), support needs would have been perceived as sufficiently met. Future studies with a modified data collection instrument would help verify this was a methodological flaw.

In Phase three focus group sessions, student's felt the severity gradation (impairment, frequency, intensity) needed to be more distinguishable across scoring options. These comments are unsurprising given that internal, emotional states can be difficult to concretely self-score and numeric scales often have arbitrary scaling, with unclear distance between answer options (78). Student feedback also allowed us to revise tool language and build-in mechanisms (e.g., hover-overs, examples) to avoid assumptions and gender- and culturally-specific references, and use person first language where possible (79). Future validation studies will confirm whether students interpret and respond to tool content as intended.

Post-secondary student mental well-being is a growing national and international priority, with recent standards calling for the integration of student-centeredness within campus mental health strategies, to ensure responsiveness to students' perceived needs and experiences (19, 25). In striving toward these principles, our work demonstrates the development of early detection capacities built for, by, and with post-secondary students. Growing research demonstrates the potential for campus-based mental health screening interventions in helping students identify unmet support needs and initiate resource-seeking (33, 35, 80). Unfortunately, measuring what matters most to end-users/patients has not been traditionally prioritized in the psychological instrument development literature (81). Diverse student engagement was a key strength of the current study. Purposive sampling allowed us to ensure focus groups reflected student voices across a range of socially co-created realities, who may have differing experiences with respect to stigma, mental health literacy, barriers to care, and systemic challenges (e.g., oppression, discrimination). Another methodological strength is our use of vignettes during Phase two expert review, allowing us to interactively engage clinicians and elicit their feedback on tool content, given they could not self-administer the tool.

We note several study limitations. Phase one discussions and outputs may have been biased by the study team's proximity to the project. However, subsequent feedback and insights from clinicians and students offered additional perspectives and opportunities to further refine HEARTSMAP-U's content. In Phase two, we did not outline clear parameters for vignette development. As a result, no vignettes evaluated the tool's scoring criteria on severe “Alcohol and drug” and “Abuse” concerns. However, clinical investigators reviewed the tool's service provisions mapping to these severe scores, and found they matched current clinical safety protocols. Additionally, we restricted focus groups to students of a single, large-size post-secondary institution in Western Canada. Students from smaller institutions (e.g., community colleges, vocational schools), rural regions, and francophone communities may see the need for further tool content modification for alignment with their experiences and learning environment (82, 83). Still, our findings may be transferable to other similarly large, research-intensive institutions.

HEARTSMAP-U has undergone a rigorous, systematic, and multi-stage tool adaptation process with clinical experts and student end-users. Later validity investigations will report evidence of HEARTSMAP-U's measurement properties, which will be crucial in gauging the tool's suitability for universal screening utility and the early detection of students' mental health needs.

## Data Availability Statement

The datasets presented in this article are not readily available because the study participants did not agree for their data to be shared publicly, due to the nature of the research. Requests to access the datasets should be directed to Punit Virk, pvirk@bcchr.ca.

## Ethics Statement

The studies involving human participants were reviewed and approved by University of British Columbia Behavioural Research Ethics Board. The patients/participants provided their written informed consent to participate in this study.

## Author Contributions

PV and QD contributed to conception and design of the study. PV and RA conducted data collection. PV, RA, and HB organized the database. PV performed the analysis and wrote the first draft of the manuscript. All authors contributed to manuscript revision, read, and approved the submitted version.

## Funding

This work was supported by The University of British Columbia Faculty of Medicine Strategic Investment Fund and The University of British Columbia Office of the Vice-President, Students. Funding sources had no involvement in study design, data collection, data analysis, writing report, or in the decision to submit for publication.

## Conflict of Interest

The authors declare that the research was conducted in the absence of any commercial or financial relationships that could be construed as a potential conflict of interest.

## Publisher's Note

All claims expressed in this article are solely those of the authors and do not necessarily represent those of their affiliated organizations, or those of the publisher, the editors and the reviewers. Any product that may be evaluated in this article, or claim that may be made by its manufacturer, is not guaranteed or endorsed by the publisher.
